# Application of Improved U-Net Convolutional Neural Network for Automatic Quantification of the Foveal Avascular Zone in Diabetic Macular Ischemia

**DOI:** 10.1155/2022/4612554

**Published:** 2022-02-26

**Authors:** Yongan Meng, Hailei Lan, Yuqian Hu, Zailiang Chen, Pingbo Ouyang, Jing Luo

**Affiliations:** ^1^Department of Ophthalmology, The Second Xiangya Hospital, Central South University, Changsha 410011, China; ^2^School of Computer Science and Engineering, Central South University, Changsha 410083, China

## Abstract

**Objectives:**

The foveal avascular zone (FAZ) is a biomarker for quantifying diabetic macular ischemia (DMI), to automate the identification and quantification of the FAZ in DMI, using an improved U-Net convolutional neural network (CNN) and to establish a CNN model based on optical coherence tomography angiography (OCTA) images for the same purpose.

**Methods:**

The FAZ boundaries on the full-thickness retina of 6 × 6 mm en face OCTA images of DMI and normal eyes were manually marked. Seventy percent of OCTA images were used as the training set, and ten percent of these images were used as the validation set to train the improved U-Net CNN with two attention modules. Finally, twenty percent of the OCTA images were used as the test set to evaluate the accuracy of this model relative to that of the baseline U-Net model. This model was then applied to the public data set sFAZ to compare its effectiveness with existing models at identifying and quantifying the FAZ area.

**Results:**

This study included 110 OCTA images. The Dice score of the FAZ area predicted by the proposed method was 0.949, the Jaccard index was 0.912, and the area correlation coefficient was 0.996. The corresponding values for the baseline U-Net were 0.940, 0.898, and 0.995, respectively, and those based on the description data set sFAZ were 0.983, 0.968, and 0.950, respectively, which were better than those previously reported based on this data set.

**Conclusions:**

The improved U-Net CNN was more accurate at automatically measuring the FAZ area on the OCTA images than the traditional CNN. The present model may measure the DMI index more accurately, thereby assisting in the diagnosis and prognosis of retinal vascular diseases such as diabetic retinopathy.

## 1. Introduction

Diabetic retinopathy (DR) is a common eye disease that affects the working population and is potentially vision- and life-threatening [1]. Macular perfusion status is important for visual function. Macular ischemia (MI) is a risk factor for DR, which leads to the destruction of the macular capillary structure, affecting treatment and prognosis [2].

The foveal avascular zone (FAZ) is a biomarker for quantifying MI in retinal vascular diseases, such as DR and retinal vein occlusion [3]. Increased size of the FAZ area may be a sign of diabetes onset and progression, providing a screening tool for assessing the progress of macular ischemia (DMI), supporting timely and effective treatment that helps prevent disease progression.

Fluorescein angiography (FA) used to be the original gold standard for FAZ measuring; however, OCTA was found to be a more reliable method in quantifying the FAZ area when compared to FA [4]. In addition, OCTA could be even superior to FA in FAZ measurement due to the fact that it is less affected by macular xanthophyll pigment shadowing by using longer wavelengths in imaging [5]. It is noninvasive, safe, and brief, and associated with excellent repeatability, and accurate determination of the FAZ area [6, 7].

However, OCTA images are detailed, complex, and contain a large volume of information, making comprehensive interpretation time-consuming and labor-intensive. Artificial intelligence technology based on deep learning can reduce the labor and cost of image acquisition, data mining, and information extraction and improve the efficiency and accuracy of disease screening, diagnosis, and retesting. A convolutional neural network (CNN) with a U-shaped structure is effective for medical image segmentation. Previous studies [8, 9] used traditional CNNs to measure the FAZ area; however, the simple jump connection between the encoder and decoder may cause information loss, and the overall position information of the segmented object in the input image may be neglected. As a result, segmentation artifacts and blurred boundaries become prominent in difficult medical image segmentation tasks, such as the FAZ measurement. The present study is aimed at improving the U-Net CNN and to apply it to the automatic measurement of the FAZ area of DMI to help obtain accurate and clinically meaningful measurements that may improve assessment efficiency.

## 2. Materials and Methods

**Figure 1:**
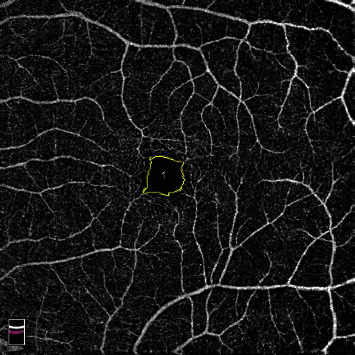
Manual marking of the full-thickness retinal FAZ using ImageJ software.

This study included patients diagnosed with DMI at the Second Xiangya Hospital of Central South University from April 2020 to April 2021. The exclusion criteria were as follows: eyes with opacity of refractive medium or other fundus diseases such as age-related macular degeneration and retinal vein occlusion. In addition, normal eyes without any fundus diseases were included. This research was approved by the Institutional Medical Ethics Board of the Second Xiangya Hospital of Central South University. All subjects underwent standard ophthalmic examination, and OCTA images were obtained using the RTVue XR Avanti (Optovue, Inc., Fremont, CA) using the AngioVue OCTA system. For OCTA images with macular edema or foveal cysts, manual correction of OCT stratification was performed first. The scanning range was 6 × 6 mm of the fovea; OCTA images that were clear and had scanning quality score of ≥6/10 were included; images that did not meet the standard were excluded. A total of 110 full-thickness retinal OCTA images were obtained. Two doctors manually marked the outline of FAZ area of the full-thickness retina by using ImageJ software (National Institutes of Health, Bethesda, MD, USA), and two senior doctors reviewed the images. For the divergent images, another retina specialist checked them and made the final decision to remove or include the image (Figure 1).

### 2.1. CNN Model Construction

**Figure 2:**
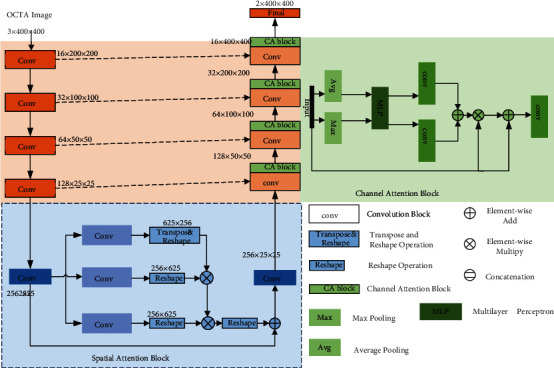
Construction of the convolutional neural network model.

In this study, an improved U-Net CNN was used to construct a new model (Figure 2). An attention mechanism was introduced into the jump connection of the traditional U-shaped structure. The baseline model was a CNN with U-Net architecture, and we added two attention modules: a spatial attention block and channel attention block.

The proposed network consisted of an encoder, a spatial attention module, decoder, and channel attention module. The basic convolution module was the basic part of the network, which was composed of a convolution layer, normalization layer, and ReLU activation layers. The encoder compressed the feature maps to obtain feature maps at different scales to extract deeper information. The encoder had four convolution modules, each of which contained two basic convolution modules and a max pooling layer. Its basic convolution module was composed of two convolution layers with a convolution kernel size of 3 × 3. The space attention block was connected after the encoder. Inspired by CS-Net [10], the basic convolution modules of the three convolution modules in the attention module were composed of a convolution layer with convolution kernel sizes of 1 × 3, 3 × 1_,_ and 1 × 1, respectively. The original feature maps obtained by the encoder were input into these three convolution modules to obtain three feature maps. The first feature map was transposed and reshaped, then the second feature map was reshaped, and then matrix multiplication was applied to fuse the information of these two feature maps. The fusion feature maps were obtained by inputting them into the Softmax layer. Similarly, the third feature map was restored to its original shape after reshaping operation and was added to the original input feature.

After the spatial attention block was the decoder, the decoder reconstructs and restores the compressed feature map, which was composed of four upsampling modules and four channel attention modules. The three basic convolution modules of the channel attention module were composed of a convolution layer with a 3 × 3 kernel size. Referring to the channel attention module of CA-Net [11], after average pooling and max pooling, the feature maps were passed through the shared fully connected layers, and convolution was performed. Afterward, the elements were element-wise added, and the matrix was multiplied by the input feature map. The input feature map was added to reduce the risk of network overfitting. Finally, the network model was composed of a convolution layer with a 1 × 1 convolution kernel size and Softmax activation layer to generate binary segmentation results.

The description data set (sFAZ) was introduced for comparison. The data set was collected by the Hong Kong Polytechnic University Eye Clinic from 45 participants, including 22 men and 23 women, aged between 18 and 49 years. The data set contained 45 eyes of the participants with 9 OCTA images per eye and a total of 405 OCTA images.

### 2.2. Data Processing

Due to the equipment and operating environment conditions, the image quality of the collected data was unstable, which affected network convergence. First, image quality may be improved by adjusting contrast, brightness, and histogram equalization. Second, we randomly divided the dataset into 70%, 10%, and 20% of the images to be used for training, verification, and testing, respectively, and fivefold crossvalidation was performed. For our dataset, we performed data enhancement operations such as rotation. For the sFAZ dataset, the image size was adjusted to 400 × 400, and data enhancement operations such as cropping and rotation were performed.

The proposed method was implemented using the PyTorch framework. We used Adaptive Moment Estimation (Adam) for the training. The initial learning rate was 0.001, the weight decay was 10^−8^, the batch size was 2, and the number of iterations was 200. The loss function we used was soft Dice loss, defined as equation (1):

(1) $$\begin{array}{c} \text{Dice loss}=1-\frac{\left|R_{p}\cap R_{g}\right|}{\left|R_{p}\right|+\left|R_{g}\right|}, \end{array}$$

where *R*_*p*_ and *R*_*g*_ represent the value of FAZ area of algorithm prediction and ground truth, respectively. The algorithm prediction means that after the training of the CNN model is completed, the model will output a predicted value of the FAZ area by inputting the original image. Ground truth refers to the real value of FAZ area that is manually marked on the original image.

The proposed method and the baseline U-Net model [12] were used to process the dataset of our study, and the accuracy of FAZ area prediction was compared between the two methods. The proposed method was applied to the public dataset sFAZ, and the evaluation index was compared with previous studies by Guo et al. [13, 14] and Liang et al. [15].

### 2.3. Model Evaluation Index

We used three indicators [13, 15, 16] to evaluate the segmentation efficiency of the model for the FAZ area. The first was the Dice score (DSC), defined by equation (2):

(2) $$\begin{array}{c} \text{DSC}=\frac{\left|R_{p}\cap R_{g}\right|}{\left|R_{p}\right|+\left|R_{g}\right|}. \end{array}$$

The second index was the Jaccard Index, defined by Equation (3):

(3) $$\begin{array}{c} \text{Jaccard}\left(R_{p},R_{g}\right)=\frac{\left|R_{p}\cap R_{g}\right|}{\left|R_{p}\right|+\left|R_{g}\right|-\left|R_{p}\cap R_{g}\right|}. \end{array}$$

The third index was the Pearson correlation coefficient, used to examine the correlation between the FAZ area predicted by the model and the ground truth, as defined in Equation (4):

(4) $$\begin{array}{c} \rho_{R_{p,R_{g}}}=\frac{\mathrm{cov}\left(R_{P},R_{g}\right)}{\sigma_{R_{p}R_{g}}}, \end{array}$$

where cov is the covariance, and *σ* is the standard deviation.

## 3. Results

**Figure 3:**
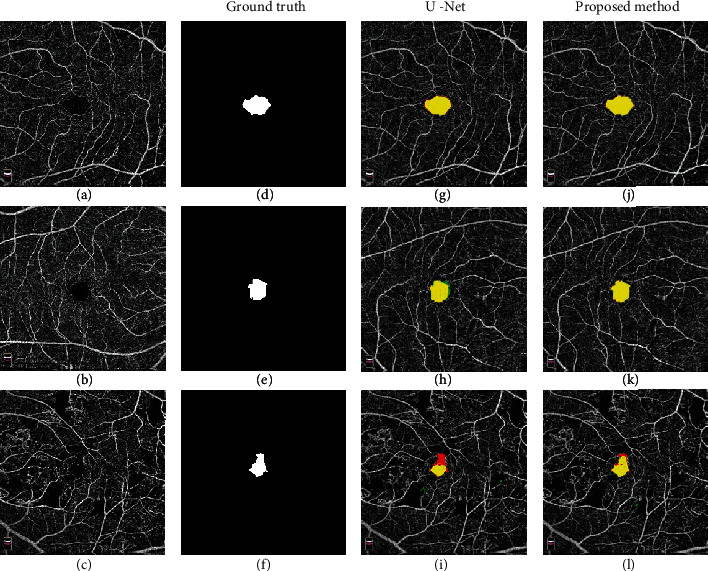
Visualization results for FAZ segmentation. (a)–(c) are the original DR map of different severity. (d)–(f) are manually labeled FAZ area as ground truth. (g)–(i) are the prediction result of traditional U-Net. (j)–(l) are the prediction result of the proposed method. The green, red, and yellow regions represent the ground truth, the predicted area, and the overlap of the two areas, respectively.

This study included 110 OCTA images, representing 88 eyes with DMI and 32 normal eyes. The DSC of the FAZ area predicted by the proposed method was 0.948 ± 0.046, the Jaccard index was 0.912 ± 0.069, and the area correlation coefficient was 0.996 ± 004. The DSC based on the baseline U-Net CNN was 0.940 ± 0.044, the Jaccard index was 0.899 ± 0.057, and the area correlation coefficient was 0.995 ± 0.004 (Table 1). In the description data set sFAZ, the DSC of the FAZ area predicted by the proposed method was 0.983 ± 0.005, the Jaccard index was 0.968 ± 0.010, and the area correlation coefficient was 0.950 ± 0.010, which were better than those previously reported in studies using the same dataset (Table 2).  Figure 3 shows the visualization results of FAZ segmentation for different degrees of DMI. The green regions of Figures 3(g)–3(l) represent the ground truth, the red regions represent the predicted FAZ area obtained by the baseline U-Net and the improved U-Net model, and the yellow regions represent the overlap between the predicted value and the real value. The larger the yellow area, the higher DSC, Jaccard, and Corr, and the smaller soft Dice loss, suggesting that the present method is more accurate than baseline U-Net for predicting the FAZ area.

## 4. Discussion

DMI affects vision. MI may help assess disease severity, visual prognosis, and treatment progress [17, 18]; consequently, it has attracted research interest. The FAZ area is an important indicator for evaluating MI. In the present study, the size of the FAZ area was highly positively correlated with the degree of DR. The higher the severity of DR classification (non-DR diabetes, nonproliferative DR, proliferative DR), the larger the FAZ area, and the greater reduction in visual acuity. The longer the onset time, the larger is the FAZ area [2, 19]. Eyes with mild to moderate nonproliferative DR had the smallest FAZ, and eyes with proliferative DR had the largest FAZ [20]. Diabetic eyes have retinal microcirculation disturbances in the macular area, which present before the onset of retinopathy, regardless of whether DR is present, and diabetic eyes show a significant increase in the FAZ area [7]. Therefore, the FAZ enlargement is considered a biomarker of DR progression [21–24]. Fluorescein fundus angiography is a standard method for diagnosing macular edema and MI. However, due to challenges associated with contrast and fluorescein leakage, the nonperfusion area of the capillary is blurred and difficult to quantify; thus, it is suboptimal for quantifying the area of MI, including the FAZ. Previous studies have used OCTA to examine changes in patients with DR, such as enlarged FAZ area, ischemic area, microaneurysms, and neovascularization [25–28]. Quantifying the capillary network of OCTA and objectively identifying the early and subtle microvascular changes in diabetic eyes to optimize the treatment of patients are an important milestone in the clinical application of OCTA.

Progress in the application of artificial intelligence to the measurement of the FAZ area of DR [29–32] has allowed to mark the FAZ area, as the first step in the present study. OCTA images contain a large amount of data, which precludes manual marking due to time and personnel requirements. The training of a CNN model requires a few accurately labeled datasets to realize the rapid positioning and segmentation of the FAZ area, providing a method suitable for use in clinical trials. The CNN greatly accelerates the speed and accuracy of medical image segmentation; thus, many researchers have used this method to measure the FAZ area [33, 34].

However, challenges associated with the use of this method remain. First, many studies on the FAZ area segmentation in OCTA images were performed in healthy subjects [35–37], while the incidence of signal noise and artifacts in OCTA imaging of diabetic patients is higher than that in their counterparts; consequently, most of the FAZ measurements in OCTA images of diabetic eyes have low accuracy [37, 38]. In medical image segmentation tasks, the most commonly used CNN model is based on U-Net [12], which consists of the contraction path of the capture context and symmetric expansion path to achieve precise positioning. The U-shape network simply combines the shallow feature map of the encoder with the deep feature map corresponding to the decoder and inputs it into the next layer. However, the cumulative stack of feature maps creates redundant information, which interferes with the network model learning useful knowledge for segmentation tasks. Guo et al. [13] used a U-Net CNN model to automatically segment and quantify the superficial FAZ area based on OCTA images and compared the automatic segmentation results with the ground truth; the maximum average DSC was 0.976, and the correlation coefficient between the area calculated by the automatic segmentation results and that calculated by the ground truth was 0.997. Liang et al. [15] evaluated two public superficial OCTA image datasets using the U-Net structure based on image reconstruction, and their DSC values were 0.9263 and 0.9784, respectively. Previous studies have shown that with the progress of network training, the distribution change of data output at each layer of the U-Net network reduced the generalization ability and training speed of the network. Traditional U-Net required development to improve the accuracy of image segmentation and automatic quantification.

For complex feature map information, although the network can provide accurate segmentation results, it is unable to determine a network feature selection strategy. Because of its poor interpretability, the CNN model is called the “black box” [39]. The attention mechanism is designed to imitate human attention, help the network focus on information that contributes greatly to the segmentation task, and visualize attention through the convolution kernel weight map of the module to enhance the interpretability of the network. The attention module has many forms, which can be divided into channel attention, spatial attention, and scale attention according to its characteristics [10]. Channel attention and spatial attention modules were used in the present study. The channel attention module selects feature maps after the fusion of the encoder and decoder, and the spatial attention module extracts position information of the segmented object from the feature map of the connection part of the decoder and encoder. The spatial attention module mainly obtains global information of the feature map through operations such as transformation and reshape and aggregates the information through matrix multiplication, thereby promoting the network to learn the location and background of the segmented objects in the feature map. The spatial attention block we use can learn the weight map of the feature according to the data feedback. It can give low weight to noise and artifacts, but give high weight to the pixels in the FAZ region. After multiplying with the original feature map, it can effectively suppress noise and artifacts. The four channel attention modules compress the connected low-level and high-level information, activate beneficial information, and suppress redundant information by data learning [11]. The segmentation results are refined by extracting the maximum connected region and filling holes. According to the obtained binary segmentation results, the area of FAZ is calculated by the pixel proportion of FAZ and the scale. In the follow-up study, the perimeter of FAZ can be calculated by counting the edge pixel value of FAZ. Using these two parameters, the roundness can be calculated by the formula of roundness, so that the FAZ area can be measured by CNN in more detail.

In this study, the channel attention and spatial attention modules were introduced into the U-Net CNN to automatically quantify the FAZ area of the DR in full-thickness retinal OCTA. The combination of the two modules effectively improved the feature selection ability of the network. In the dataset we collected, the best DSC was 0.981, which was better than that in previous studies based on U-Net [12]. In the public dataset sFAZ, the DSC of the proposed method was 0.948, which was better than those previously reported by Guo et al. and Liang et al. [13–15].

In the measurement of the FAZ area, some previous studies [24] only detected the superficial retinal capillary plexus (SCP) or deep retinal capillary plexus (DCP) index; however, for the determination of MI, a single-layer index is not accurate. Therefore, we measured the FAZ area of the full-thickness retina, which can more comprehensively reflect the real macular perfusion state. Arya et al. [40] found that measurements on DCP or SCP are susceptible to segmentation errors, while vascular measurements on the full-thickness retina have higher repeatability. Because different OCT instruments have different retinal layering algorithms for the retina assessment, the definitions of the start and end points of the SCP and DCP are different, which precludes the analysis the OCT and OCTA images obtained by different devices in a unified manner [41–44]. The start and end points of the full-thickness retina measurements are essentially the same for different devices. Therefore, Custo et al. [23] believed that full-thickness retinal OCTA images are repeatable and reproducible in the eyes with or without diabetic macular edema and using different devices, and that it is more straightforward and accurate to reproduce them in clinical and ophthalmic reading centers.

Furthermore, the built-in software of the OCTA instrument is more accurate in measuring the FAZ area of normal eyes, but for patients with macular edema in the DR, the OCTA layering deviation may be caused by the edema, which affects the determination of OCTA images by the CNN model. Sorour et al. [45] thought that using the default full retinal projection may lead to the erroneous segmentation duo to the existence of DME, as the DME has little effect of the RPE; so, they customized the lower boundary of the full retina slab according to the RPE to get more accurate results. Reza et al. [9] manually adjusted the automatic stratification of OCT and found that it could effectively reduce the large error caused by the automatic measurement of built-in software. Therefore, in our study, we adopted a strategy of manually adjusting OCT stratification for OCTA images with macular edema or foveal cysts.

This study had several limitations. First, because some DMI patients have concomitant cataracts and/or severe diabetic macular edema, low-quality OCTA images were excluded from this study; the sample size was small, and the grading of different DR degrees was not performed, which limits the learning ability of the CNN model. In future research, increasing the sample size and making a more detailed division may produce more powerful results; second, in addition to DR, MI will appear in many ischemic retinopathies; future studies should account for more diseases and OCTA indicators to improve the automatic quantitative accuracy of a model for MI.

## 5. Conclusions

The present study has proposed a new CNN model to automatically measure the FAZ area of the DMI, which performed better than did the baseline U-Net model. The present model may improve the diagnosis and prognosis of MI in retinal diseases.

## Figures and Tables

**Table 1:** Processing results of the proposed method and baseline U-Net on our research dataset.

	Dice score	Jaccard index	Area Corr
Baseline (U-Net)	0.940; 0.044	0.899; 0.057	0.995; 0.004
Proposed method	0.948; 0.046	0.912; 0.069	0.996; 0.004

**Table 2:** Results of the proposed method and other studies on SFAZ dataset.

	Dice score	Jaccard index	Area Corr
2019 Guo et al. [13]	0.976, -	—	0.997, -
2020 Guo et al. [14]	0.920, 0.030	—	—
2021 Liang [15]	0.978, 0.002	0.958, 0.003	0.999, 0.001
Proposed method	0.983, 0.005	0.968, 0.010	0.950, 0.010

## Data Availability

Data are available upon request from authors.
